# Hematopoietic Stem/Progenitor Cells and the Pathogenesis of HIV/AIDS

**DOI:** 10.3389/fcimb.2020.00060

**Published:** 2020-02-21

**Authors:** Tetsuo Tsukamoto

**Affiliations:** Department of Immunology, Faculty of Medicine, Kindai University, Osaka, Japan

**Keywords:** human immunodeficiency virus, acquired immunodeficiency syndrome, hematopoietic stem/progenitor cells, hematopoiesis, senescence

## Abstract

The interaction between human immunodeficiency virus (HIV) and hematopoietic stem/progenitor cells (HSPCs) has been of great interest. However, it remains unclear whether HSPCs can act as viral reservoirs. Many studies have reported the presence of latently infected HSPCs in the bone marrow of HIV-infected patients, whereas many other investigators have reported negative results. Hence, further evidence is required to elucidate this controversy. The other arm of HSPC investigations of HIV infection involves dynamics analysis in the early and late stages of infection to understand the impact on the pathogenesis of acquired immunodeficiency syndrome. Several recent studies have suggested reduced amounts and/or functional impairment of multipotent, myeloid, and lymphoid progenitors in HIV infection that may contribute to hematological manifestations, including anemia, pancytopenia, and T-cell depletion. In addition, ongoing and future studies on the senescence of HSPCs are expected to further the understanding of HIV pathogenesis. This mini review summarizes reports describing the basic aspects of hematopoiesis in response to HIV infection and offers insights into the association of HIV infection/exposure of the host HSPCs and hematopoietic potential.

## Introduction

Human immunodeficiency virus (HIV) infection causes acquired immunodeficiency syndrome (AIDS). The depletion of memory CD4^+^ T cells preceding the manifestation of AIDS may be mainly due to HIV infection of these cells. However, HIV may also cause reduced production of naïve T cells by infection of CD4^+^ thymocytes. Although the dynamics of hematopoietic stem/progenitor cells (HSPCs) in response to HIV infection remains unclear, it is well-established that HIV infection is associated with hematological changes, such as anemia and pancytopenia (Parinitha and Kulkarni, 2012; Durandt et al., 2019). Therefore, it is imperative to better elucidate the contribution of altered hematopoietic potential to the disease. The aim of this mini review was to discuss on factors affecting the physiology and pathology of HSPCs by reviewing past publications describing the interactions between HIV and hematopoietic progenitor cells (HPCs) in the bone marrow (BM) and thymus for better understanding the role of hematopoiesis in the pathogenesis.

## HSPCs in the BM

**Figure 1:**
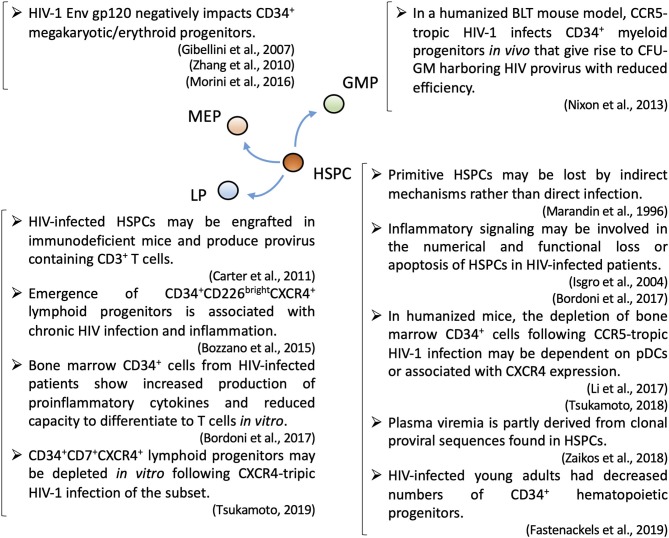
A summary of recent topics regarding HIV-1 pathogenesis associated with subsets of hematopoietic stem/progenitor cells. CFU-GM, granulocyte-macrophage colony-forming unit; GMP, granulocyte-macrophage progenitor; HSPC, hematopoietic stem progenitor cell; LP, lymphoid progenitor; MEP, megakaryocyte–erythrocyte progenitor; pDC, plasmacytoid dendritic cell.

Adult hematopoietic differentiation occurs in the BM. Hematopoietic stem cells (HSCs) have long-term self-renewing capacity and can differentiate to any type of blood cell (Rieger and Schroeder, 2012). Although HSC niches have not been fully defined (Morrison and Scadden, 2014), a recent study indicated that HSCs reside in a perivascular niche and are supported by various cytokines secreted by endothelial and stromal cells (Ding et al., 2012). BM HSPCs consist of progenitors for all blood cell lineages including those described in Figure 1 (Rieger and Schroeder, 2012). Proteomic and transcriptomic analyses have reported many potential factors that may work in concert in hematopoiesis, although the significance of individual genes must be further clarified (Liu et al., 2006; Kim et al., 2009; Starnes et al., 2010). Recent evidence indicates that inflammatory signals, such as prostaglandin E2, nitric oxide, granulocyte colony-stimulating factor, interferons, tumor necrosis factor, and Toll-like receptor 4, may be involved in the emergence of HSPCs (He et al., 2015; Luis et al., 2016).

## The Roles of HSPCs in T-Lineage Differentiation

T-lineage differentiation in the thymus is dependent on a supply of CD34^+^ progenitors from the BM (Kondo et al., 1997). Early lymphoid progenitors (LPs) are thought to reside in distinct niches from those of HSCs (Ding and Morrison, 2013). CD34^+^CD38^dim^, but not CD34^+^CD38^+^, cells can migrate to the thymus and commit to the T cell lineage (Res et al., 1996). Transcriptional regulation of the lymphoid commitment of HSPCs is complex (Laurenti et al., 2013). Notch 1 and its ligands play essential roles in T-lineage commitment (Radtke et al., 2004). For example, Delta-like 1 (DL1) enhances the repopulation capability of human CD34^+^CD38^−^ cells in the BM and contributes to the generation of thymus-repopulating T-cell precursors (Ohishi et al., 2002). In addition, Delta-like 4 (DL4) induces Notch signaling in the thymus (Hozumi et al., 2008). Accordingly, the stable expression of DL1 or DL4 by OP9 cells allows for the differentiation of human HSPCs to T cells *in vitro* (La Motte-Mohs et al., 2005; Mohtashami et al., 2010). C-X-C chemokine receptor type 4 (CXCR4) also plays a critical role in the localization and differentiation of T-lineage progenitors in the thymus (Plotkin et al., 2003).

## HSPC-Associated Hematological Changes in HIV Infection

Hematological changes in HIV-infected patients may be at least partly associated with abnormalities in the BM (Dhurve and Dhurve, 2013; Durandt et al., 2019). Because HSPCs generally have limited surface expression of CD4, their abnormalities in HIV infection could be largely explained as an indirect effect of HIV infection, rather than the results of direct infection of HSPCs (Louache et al., 1992; De Luca et al., 1993; Maciejewski et al., 1994; Marandin et al., 1996; Koka et al., 1999). Although antiretroviral therapy (ART) generally improves hematopoiesis in HIV-infected patients (Baillou et al., 2003), the immune function in some patients is insufficient despite successful ART; therefore, such patients are referred to as immunological non-responders (Corbeau and Reynes, 2011; Takuva et al., 2014; Rb-Silva et al., 2019). Indeed, the recovery of CD4^+^ T cell counts after successful ART may depend on the recovery of CD34^+^ cell counts (Sauce et al., 2011).

Lymphopoiesis, myelopoiesis, megakaryopoiesis, and erythropoiesis may be altered during the course of HIV infection (Figure 1). HIV-1 infection may cause defective myelopoiesis/erythropoiesis as well as the accumulation of myeloid/erythroid precursors (Costantini et al., 2009, 2010). Ineffective platelet production noted in HIV-infected patients (Cole et al., 1998) might be due to a negative impact of HIV on the differentiation of megakaryocyte lineages, leading to thrombocytopenia (Costantini et al., 2006; Sundell and Koka, 2006). The V3 loop region of the HIV-1 gp120 envelope protein was described as a potential inhibitor of megakaryocyte differentiation (Zhang et al., 2010). Furthermore, studies have suggested the influence of HIV-1 gp120/CD4 interaction on CD34^+^ megakaryocytic/erythroid progenitors (Gibellini et al., 2007; Morini et al., 2016).

## The Biological Functions of HIV Coreceptors

HIV-1 uses C–C chemokine receptor type 5 (CCR5) and CXCR4 as coreceptors (Weiss, 1996). CCR5 is expressed on the surface of memory CD4^+^ T cells and causes the massive depletion of this cell type following HIV-1 infection of the host (Mattapallil et al., 2005). Recent evidence suggests that CCR5 is involved in inflammation (Kitade et al., 2012; Barashi et al., 2013; Duan et al., 2014) because the lack of a functional CCR5 allele is associated with the severity of viral infection, possibly due to altered immune responses (Lim et al., 2008). On the other hand, the pathological roles of CCR5 in various infectious and non-infectious diseases, e.g., autoimmune diseases, have been suggested (Vangelista and Vento, 2017). For example, the depletion of CCR5 was associated with attenuation of the adverse effects of inflammation (Muntinghe et al., 2009), and blockade of CCR5 inhibited leukocyte trafficking and reportedly reduced inflammation in a murine model of colitis (Mencarelli et al., 2016). Thus, these findings address the roles of CCR5 in health and disease.

CXCR4 is specific for stromal cell-derived factor 1 (SDF-1, also known as CXCL12). SDF-1 is produced by BM stromal cells, including CXCL12-abundant reticular cells (Nagasawa, 2015), and allows the homing of HSCs to BM. The interaction between SDF-1 and CXCR4 is essential for hematopoiesis (Karpova and Bonig, 2015). In addition, the SDF-1/CXCR4 axis has multiple essential roles in life (Murphy and Heusinkveld, 2018), such as embryonic (Mcgrath et al., 1999) and vascular (Takabatake et al., 2009; Kim et al., 2017) development, while providing support for the survival and migration of neoplastic cells (Chatterjee et al., 2014). The polymorphisms of SDF-1 might affect the ability to prevent HIV-1 infection (Winkler et al., 1998; Kuipers et al., 1999). However, the effect of SDF-1 polymorphisms on the susceptibility of the host to HIV-1 infection might be moderate (Ding et al., 2018). In contrast to the popularity of the topics of CXCR4 as an HIV-1 coreceptor and SDF-1 as an inhibitor of HIV-1 infection (Arenzana-Seisdedos, 2015), relatively few articles have addressed the intrinsic functions of SDF-1 and CXCR4 in the pathogenesis of HIV-1 infection and AIDS (Ikegawa et al., 2001; Tsukamoto, 2018).

## Potential Mechanisms Underlying the Loss of or Changes in HSPCs in Response to HIV Infection of the Host

Various potential mechanisms underlying changes in HSPCs during HIV infection have been suggested, such as reduced c-Mpl (thrombopoietin receptor) expression on HSPCs (Koka et al., 2004), elevated plasma SDF-1 levels (Ikegawa et al., 2001), and altered BM niches (Moses et al., 1996). HIV-1 infection results in increased levels of inflammatory cytokines, affecting dynamics and functions (Bordoni et al., 2017) or inducing Fas-mediated apoptosis (Isgro et al., 2004) of HSPCs (Figure 1). Importantly, HSPCs require inflammatory signals in their development (Luis et al., 2016), and therefore may contribute to inflammation (Fischer and Agrawal, 2013). A recent study reported the emergence of a CD34^+^CD226(DNAM-1)^bright^CXCR4^+^ LP subset in association with chronic HIV infection and inflammation, reflecting altered dynamics of natural killer (NK) cells and α/β T cells (Bozzano et al., 2015; Figure 1). Finally, there has recently been an emerging trend to interpret some hematopoietic changes during the course of HIV infection as the accelerated senescence of HSPCs (Appay and Sauce, 2017; Fali et al., 2018; Fastenackels et al., 2019).

Humanized mouse models provide important resources for the analysis of BM HSPCs following HIV-1 infection. For example, in studies with humanized mice challenged with CXCR4-tropic HIV-1_NL4−3_, CD34^+^ cells were depleted and/or exhibited impaired *ex vivo* myeloid and erythroid colony-forming capacities (Jenkins et al., 1998; Koka et al., 1998). Moreover, the reduction in BM HSPC counts in humanized mice was observed even after CCR5-tropic HIV-1 infection (Arainga et al., 2016). Other research groups have reported that the loss of CD34^+^ cells in CCR5-tropic HIV-1 infection might be dependent on plasmacytoid dendritic cells (pDCs) (Li et al., 2017) or correlated with CXCR4 expression (Tsukamoto, 2018; Figure 1). Therefore, it is important to further investigate changes such as altered expression of cytokines in pDCs and other cells residing in BM in HIV infection. The latter could implicate the involvement of SDF-1/CXCR4 axis in the pathogenesis such as accelerated turnover of HSPCs.

## The Impact of HIV on T-Lineage Development

The involvement of the thymus in HIV pathogenesis has been investigated (Ye et al., 2004). HIV-1 may cause thymocyte depletion mediated by an indirect cytopathic effect and infection of CD3^−^CD4^+^CD8^−^ progenitor cells (Su et al., 1995). In an *in vitro* model imitating the thymic environment, thymocyte maturation was inhibited by HIV infection of the CD44^+^CD25^−^CD3^−^ cell lineage (Knutsen et al., 1999). Early ART might preserve the lymphopoiesis capability of the host (Bordoni et al., 2015b, 2018; Rb-Silva et al., 2019) and reverse reduced thymic function (Withers-Ward et al., 1997; Levine et al., 2001).

In a BLT (BM, liver, and thymus) mouse model, HIV-1 Nef enhanced HIV-1 replication and caused depletion of CD4^+^CD8^+^ thymocytes (Zou et al., 2012). In another humanized mouse model, HIV-1 infection caused perturbation of cytokine mRNA expression in infected thymocytes. For instance, mRNA levels of interleukin (IL)-6, interferon-γ, and IL-2 were increased, whereas macrophage inflammatory protein (MIP)-1β expression was decreased. On the other hand, HIV infection of human stromal cells increased IL-6 levels, whereas SDF-1 expression levels were unaffected (Koka et al., 2003).

There have also been several reports on the T-lineage differentiation of HPCs, although it may be difficult to interpret all the data collectively. T-lineage progenitors express CXCR4 and are susceptible to CXCR4-tropic HIV infection (Berkowitz et al., 1998). In a study, BM cells infected with HIV before ART initiation had reduced amounts of CD34^+^ cells, but not CD34^+^CD7^+^ LPs (Muller et al., 2002). Although the data are intriguing, the study lacked information of absolute cell counts, so their notions were not firmly concluded except for reduced CD34^+^ frequencies. In another study of BM samples from HIV-infected ART-treated immunological non-responders, clonogenic capability and the sizes of primitive HSPCs were altered, which were associated with reduced production of IL-2, increased production of TNF-α, and increased stromal production of IL-7 (Isgro et al., 2008). Another study using a lentiviral vector expressing HIV-1 Nef showed that Nef may impair the differentiation of HSPCs to CD3ε^+^CD5^+^CD1a^+^ T/NK precursors (Dorival et al., 2008).

In a recent study, BM-derived HSPCs from HIV-infected patients exhibited reduced T-cell differentiation potential and increased production of pro-inflammatory cytokines, indicating that they are also produced by non-LPs. However, it remains unclear whether pro-inflammatory cytokine secretion is the cause or consequence of impaired T cell differentiation potential (Bordoni et al., 2017; Figure 1). Also, in a macaque model, following challenge with simian immunodeficiency virus, BM-derived CD34^+^ cells exhibited reduced T-lineage differentiation potential *in vitro* without significant changes in phenotypic analysis of CD34^+^ subsets (Thiebot et al., 2005). Another recent study suggested that CD34^+^CD7^+^CXCR4^+^ cells may be depleted in response to CXCR4-tropic HIV-1 infection in a coculture of HIV-infected umbilical cord-derived CD34^+^ and OP9-DL1 cells (Tsukamoto, 2019b; Figure 1). Despite the evidence of LPs during HIV-1 infection, our understanding of the impact of HIV-1 on LPs remains limited.

## Direct HIV Infection of HSPCs

HSPCs have limited surface levels of HIV receptors and coreceptors compared with differentiated CD4^+^ cells. CD34^+^CD133^+^ umbilical cord-derived HSCs may have further limited expression levels of CD4, CXCR4, and CCR5 (Hariharan et al., 1999). In an *in vitro* culture study, BM-derived CD34^+^CD38^−^ primitive HPCs were exposed to HIV-1 or HIV-2, but infection was not observed (Weichold et al., 1998). In another study, HIV-1 exposure had no effect on the *in vitro* expansion/proliferation dynamics of HSPCs (Kaushal et al., 1996). However, accumulating evidence has implicated HIV-susceptible subsets of HSPCs in patients (Louache et al., 1994; Zauli et al., 1994; Chelucci et al., 1995, 1999). In addition, peripheral blood CD34^+^ cells expressing CXCR4/CCR5 are susceptible to diverse strains of HIV-1 (Ruiz et al., 1998). Another study found that BM CD34^+^CD4^+^ cells are depleted in HIV-infected patients (Banda et al., 1999). Moreover, the HIV-1 Gag protein was expressed by BM HSPCs isolated from HIV-infected patients (Carter et al., 2010). A recent study of patient samples revealed that some HSPC subsets express high levels of CD4 and may harbor both CCR5-tropic and CXCR4-tropic HIV genomes (Sebastian et al., 2017). Furthermore, HSPCs latently infected with cytomegalovirus may have enhanced susceptibility to HIV-1 infection (Cheung et al., 2017). To confirm this evidence, another study using humanized BLT mice demonstrated HIV-1 infection of HPCs *in vivo*. These infected HPCs remained capable of differentiating to myeloid cells *in vitro*, albeit with reduced efficacy (Nixon et al., 2013; Figure 1).

Regarding molecular mechanisms preventing HIV infection except limited (co) receptor expression, a recent study suggested a post-entry mechanism to allow HSPCs to restrict HIV-1 replication prior to conversion of viral RNA into DNA and integration into the host genome (Griffin and Goff, 2015). Variations of tripartite motif-containing protein 5 may also influence the infection efficiency of lentiviruses in human and rhesus HSPCs (Evans et al., 2014). CCR5-ligand β-chemokines, including RANTES and MIP-1β, produced by HSPCs may modify the susceptibility of these cells to CCR5-tropic HIV-1 Env (Majka et al., 1999, 2000).

Some studies on HIV infection of HSPCs have relied on *in vitro* stimulation of cells with 50–100 ng/mL of individual stem cell factors, thrombopoietin, or FMS-like tyrosine kinase 3 ligand to overcome the low permissiveness of these cells to retrovirus/lentivirus infection (Santoni De Sio and Naldini, 2009). Such stimulation may enhance gene expression of the HIV-1 receptor and coreceptors, leading to overestimation of HIV infection/replication levels in HSPCs (Zhang et al., 2009). A method to achieve reproducible *in vitro* infection of HSPCs with CXCR4-tropic HIV-1 with RetroNectin-coated plate, but without strong cytokine stimulation, has been proposed (Tsukamoto and Okada, 2017).

## HSPCs as Viral Reservoirs

There is no consensus on whether HSPCs are a major HIV reservoir (Von Laer et al., 1990; Stanley et al., 1992; Neal et al., 1995; Kandathil et al., 2016). A relatively recent study of BM HSPCs from eight patients following long-term effective ART found no HIV DNA in the collected cells (Josefsson et al., 2012), suggesting that HIV reservoir surveys of purified CD34^+^ cells may fail to exclude HIV-contaminated CD4^+^ T cells (Durand et al., 2012). In contrast, accumulating data support latent HIV infection of HSPCs. Moreover, some BM HSPCs may remain latently infected after successful treatment (Bordoni et al., 2015a). Another study suggested that multiple subsets of HSPCs may be latently infected with HIV-1, including immature (CD34^+^CD38^−^CD45RA^−^) progenitors, which are more likely to persist and serve as latent reservoirs following ART (Mcnamara et al., 2012). Humanized mouse models have also been utilized to investigate CD34^+^ HIV reservoirs. A previous study revealed that the HIV-infected HSPCs may serve as long-term HIV reservoirs in the BM of humanized mice, leading to production of HIV-integrated CD3^+^ T cells (Carter et al., 2011). Taken together, HSPCs might constitute significant HIV reservoirs, which should be further investigated.

While it remains unclear whether infected HSPCs contribute to residual viremia after ART (Onafuwa-Nuga et al., 2010; Mcnamara and Collins, 2011), a recent article reported that HSPCs in suppressed patients harbor functional HIV proviral genomes that often match residual peripheral viral RNA (Zaikos et al., 2018). If these findings are confirmed, HSPCs might be finally regarded as long-term viral reservoirs, because they are long-lived cells with regulated susceptibility to apoptosis (Durdik et al., 2017). Thus, precise identification of HSPC subsets harboring functional HIV proviral copies could further facilitate these findings and clarify the role of HSPCs in HIV persistence even after successful ART. Furthermore, it is interesting to assess whether early initiation of ART could prevent the establishment of viral reservoirs in HSPCs.

## Protection of HSPCs Against HIV Infection

Presently, the best method for treating HIV-infected individuals in terms of protection of HSPCs is to initiate ART as early as possible regardless of the disease stage (World Health Organization, 2015). By interrupting HIV pathogenesis early during infection, it is expected that existing CD4^+^ T cells and HSPCs as well as the host's hematopoietic capacity will be preserved for long (Bordoni et al., 2015b). However, more treatment options might be helpful for patients who are diagnosed in the chronic phase and/or those who manifest the characteristics of immunological non-responders against the current ART regimens (Rb-Silva et al., 2019).

CXCR4 may be targeted to protect HSPCs against CXCR4-tropic HIV-1 infection, because they express CXCR4 and are considered susceptible to CXCR4-tropic HIV-1 infection. For example, the μ-opioid agonist DAMGO (C_26_H_35_N_5_O_6_) was found to downregulate CXCR4 expression and prevent HIV-1 infection of BM HSPCs (Strazza et al., 2014). On the other hand, a clinical study reported that the CXCR4 antagonist plerixafor was not successful for the treatment of HIV-infected patients (Hendrix et al., 2004). Because systemic administration of plerixafor is associated with adverse effects, especially to patients with cardiovascular diseases, further development of CXCR4-tropic HIV-1 entry inhibitors with weaker affinity to CXCR4 than plerixafor is needed (Berg et al., 2018). Other entry inhibitors such as ibalizumab, a humanized monoclonal anti-CD4 antibody that inhibits the binding of HIV gp120, might also be highly effective in preventing HSPCs from infection (Emu et al., 2018). It is unclear whether CCR5-tropic HIV-1 entry inhibitors such as maraviroc are effective in protecting HSPCs because HIV-1 is considered to use CXCR4 to enter those cells (Carter et al., 2011). However, those entry inhibitors can lower viral burden by protecting CCR5^+^ memory CD4^+^ T cells and lead to lower risks for indirect damages to HSPCs.

The significance of CXCR4 in HIV-1 infection is not necessarily limited to its function as an HIV-1 coreceptor. For example, it is unclear how the biological roles of CXCR4, including the SDF-1/CXCR4 signaling pathway in the BM and thymus, affect hematopoiesis in response to HIV infection. It has been indicated that elevation of plasma SDF-1 levels may be associated with disease progression (Ikegawa et al., 2001). Another study suggested the use of granulocyte colony-stimulating factor to increase CD34^+^ and CD4^+^ cell counts in HIV-infected patients (Nielsen et al., 1998). In addition, a recent humanized mouse study indicated the involvement of CXCR4 in the loss of BM HSPCs in CCR5-tropic HIV-1 infection (Tsukamoto, 2018; Figure 1). These results must be further investigated to elucidate whether the loss of HSPCs following HIV-1 infection can be alleviated by interrupting the SDF-1/CXCR4 signaling pathway.

Recent studies indicate HSPCs as an ideal target for anti-HIV gene therapy aimed to protect hosts' hematopoietic potential (Kitchen et al., 2011; Savkovic et al., 2014). For detailed discussions on recent advances in the field, see a recently published review by this author Tsukamoto (2019a).

## Concluding Remarks

Despite previous efforts and accumulating data to better clarify the interactions between HIV-1 and HSPCs, studies on their involvement in HIV pathogenesis are ongoing. The contribution of latently infected HSPCs to viral persistence should be better described. Regarding HSPC subsets, recent evidence supports the influence of HIV-1 on myeloid progenitor cells. On the other hand, among various steps in T-lineage development, the functional and numerical alteration of CD34^+^ LPs in HIV-1 infection needs to be further elucidated to improve the current understanding of the degree of impaired CD4^+^ T-cell generation on peripheral CD4^+^ T-cell loss and AIDS onset. Humanized mouse models and *in vitro* models including OP9-DL1/OP9-DL4 coculture systems could be used for further analysis of HSPCs in HIV infection in this context. Further investigations in these fields will collectively enhance our understanding on the significance of protecting HSPCs in HIV infection.

## Author Contributions

The author confirms being the sole contributor of this work and has approved it for publication.

### Conflict of Interest

The author declares that the research was conducted in the absence of any commercial or financial relationships that could be construed as a potential conflict of interest.
